# *N*,*N*′-Bis(5-bromo­pyridin-2-yl)methane­diamine

**DOI:** 10.1107/S160053681100821X

**Published:** 2011-03-09

**Authors:** Gary S. Nichol, Anuj Sharma, Hong-Yu Li

**Affiliations:** a Department of Chemistry and Biochemistry, 1306 E University Boulevard, The University of Arizona, Tucson, AZ 85721, USA; b Southwest Center for Drug Discovery, College of Pharmacy, The University of Arizona, Tucson, AZ 85737, USA

## Abstract

The V-shaped title compound, C_11_H_10_Br_2_N_4_, lies on a crystallographic twofold rotation axis which passes through the central C atom. In the crystal, an infinite tape motif, which propagates in the *a*-axis direction, is formed by inversion-related N—H⋯N hydrogen-bonding inter­actions. The structure confirmed the identity of the compound as a reaction side product.

## Related literature

For background information on the Groebke–Blackburn synthesis, see: Bienaymé & Bouzid (1998 ▶); Blackburn *et al.* (1998 ▶); Groebke *et al.* (1998 ▶); Mandair *et al.* (2002 ▶); Parchinsky *et al.* (2006 ▶). For the crystal structure of a similar compound, see: Wu *et al.* (2004 ▶). For information on graph-set notation to describe hydrogen-bonding motifs, see: Bernstein *et al.* (1995 ▶).
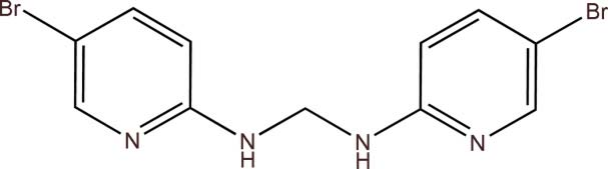


## Experimental

### 

#### Crystal data

C_11_H_10_Br_2_N_4_
*M* *_r_* = 358.05
Monoclinic, 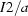

*a* = 11.9075 (6) Å
*b* = 4.0523 (2) Å
*c* = 25.8065 (15) Å
β = 98.326 (3)°
*V* = 1232.11 (11) Å^3^
*Z* = 4
Mo *K*α radiation
μ = 6.56 mm^−1^
*T* = 100 K
0.24 × 0.08 × 0.07 mm

#### Data collection

Bruker Kappa APEXII DUO CCD diffractometer
Absorption correction: numerical (*SADABS*; Sheldrick, 1996 ▶) *T*_min_ = 0.297, *T*_max_ = 0.675
11800 measured reflections
1903 independent reflections
1674 reflections with *I* > 2σ(*I*)
*R*_int_ = 0.023

#### Refinement

*R*[*F* ^2^ > 2σ(*F* ^2^)] = 0.019
*wR*(*F* ^2^) = 0.051
*S* = 1.03
1903 reflections
98 parameters
1 restraint
H atoms treated by a mixture of independent and constrained refinement
Δρ_max_ = 0.70 e Å^−3^
Δρ_min_ = −0.47 e Å^−3^

### 

Data collection: *APEX2* (Bruker, 2007 ▶); cell refinement: *SAINT* (Bruker, 2007 ▶); data reduction: *SAINT*; program(s) used to solve structure: *SHELXTL* (Sheldrick, 2008 ▶); program(s) used to refine structure: *SHELXTL*; molecular graphics: *ORTEP-3 for Windows* (Farrugia, 1997 ▶); software used to prepare material for publication: *SHELXTL*, *publCIF* (Westrip, 2010 ▶) and local programs.

## Supplementary Material

Crystal structure: contains datablocks I, global. DOI: 10.1107/S160053681100821X/ng5123sup1.cif

Structure factors: contains datablocks I. DOI: 10.1107/S160053681100821X/ng5123Isup2.hkl

Additional supplementary materials:  crystallographic information; 3D view; checkCIF report

## Figures and Tables

**Table 1:** Hydrogen-bond geometry (Å, °)

*D*—H⋯*A*	*D*—H	H⋯*A*	*D*⋯*A*	*D*—H⋯*A*
N2—H2*N*⋯N1^i^	0.87 (1)	2.11 (1)	2.9645 (18)	168 (2)

Symmetry code: (i) 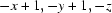.

**Fig. 1.**
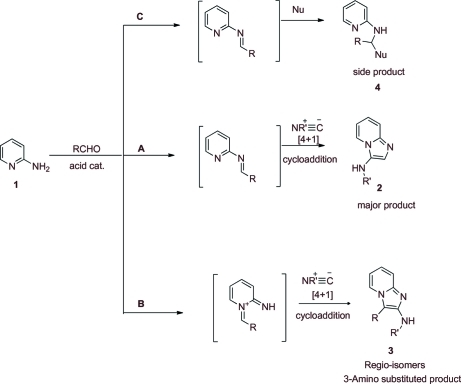
Three reaction pathways in the Groebke-Blackburn reaction.

**Fig. 2.**
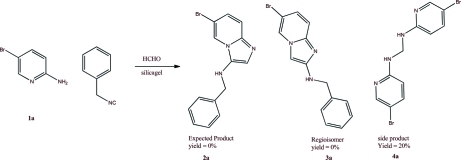
Three possible products in the reaction described herein.

**Fig. 3.**
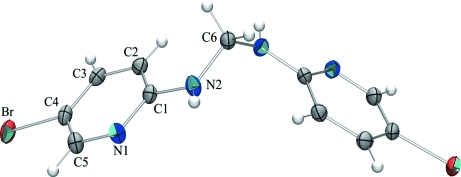
The molecular structure of 4a, with displacement ellipsoids at the 50% probability level. Unlabelled atoms are related to labelled atoms by a twofold rotation (symmetry operator: -x + 3/2, y, -z).

**Fig. 4.**
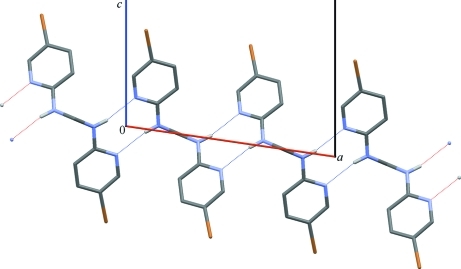
Hydrogen bonding interactions (blue dotted lines; red dotted lines indicate continuation) in the crystal structure of 4a. The long c axis has been truncated.

## supplementary crystallographic information

### Comment

The Groebke-Blackburn reaction is the most popular way to prepare imidazo-azines from 2-aminoazines in a single-step. (Groebke *et al.*, 1998; Bienaymé & Bouzid,1998; Blackburn *et al.*, 1998). The reaction involves addition of 2-aminoazine 1 to the aldehyde in the presence of catalytic amounts of acid to generate the respective Schiff base which undergoes a non-concerted [4 + 1] cycloaddition with an isocyanide to form the imidazoazine 2 (Pathway A, Figure 1). Though imidazoazine 2 remained the major product, it was later found that the reaction also produced the isomeric imidazo[1,2-*a*]pyrimidine product 3 through an alternative iminium intermediate involving the ring nitrogen of 1 (Pathway B, Figure 1; Parchinsky *et al.*, 2006). Similarly, nucleophilic solvents (for example, methanol) were found to promote interaction of the primary imine intermediate with the second molecule of 2-aminoazine or the solvent itself to give side-products like 4 (Pathway C, Figure 1; Mandair *et al.*, 2002).

We, in one case, decided to synthesize *N*-benzyl-6-bromoindolizin-3-amine, 3a. Interestingly, the reaction did not yield the expected product 2a or the regioisomer 3a. However, the product which crystallized from a solution of dichloromethane turned out to be *N*,*N'*-bis(5-bromopyridin-2-yl)methanediamine, 4a in 20% yield (Figure 2).

The V-shaped structure of 4a is shown in Figure 3. The compound has crystallized with atom C6 on a twofold rotation axis, and has been set in space group *I*2/*a*. Molecular dimensions are unexceptional. In the crystal, inversion-related N—H···N hydrogen bonding interactions form an *R*^2^_2_(8) graph set motif (Bernstein *et al.*, 1995). As a result, the crystal forms an infinite hydrogen bonded tape of V-shaped molecules, which propagates in the *a*-axis direction. The tapes are stacked in the *b*-axis direction, and the separation between each tape is approximately 3.6 Å. The structure of the related compound *N*,*N'*-Di-2-pyridylmethylenediamine exhibits the same V-shaped structure, but with a different crystal packing arrangment (Wu *et al.*, 2004).

### Experimental

To a solution of 5-bromopyridin-2-amine 1a (0.58 mmol, 100 mg) in dichloromethane (DCM) (1.5 ml), was added aq. 37% solution of formaldehyde (140 µl, 1.78 mmol) followed by (isocyanomethyl)benzene (75.4 µl, 0.58 mmol) and the solution was stirred for 10 min. DCM was evaporated and the resulting solid was irradiated under microwave at 100° C for 10 min. The crude product was purified through silica gel chromatography to provide 41 mg of 4a in (20% yield). The product was recrystallized from a DCM solution.

### Refinement

All H atoms were located in a difference map and are freely refined, with the exception of an N–H distance restaint of 0.88 (1) Å used on H2N. C–H distances lie in the range 0.92 (2) to 1.01 (2) Å.

The space group was set as I2/a since I2/a results in a smaller beta angle (and slightly shorter c axis).

### Crystal data

**Table:**

C_11_H_10_Br_2_N_4_	*F*(000) = 696
*M**_r_* = 358.05	*D*_x_ = 1.930 Mg m^−^^3^
Monoclinic, *I*2/*a*	Mo *K*α radiation, λ = 0.71073 Å
Hall symbol: -I 2ya	Cell parameters from 6689 reflections
*a* = 11.9075 (6) Å	θ = 3.2–30.6°
*b* = 4.0523 (2) Å	µ = 6.56 mm^−^^1^
*c* = 25.8065 (15) Å	*T* = 100 K
β = 98.326 (3)°	Rod, colourless
*V* = 1232.11 (11) Å^3^	0.24 × 0.08 × 0.07 mm
*Z* = 4	

### Data collection

**Table:**

Bruker Kappa APEXII DUO CCD diffractometer	1903 independent reflections
Radiation source: fine-focus sealed tube with Miracol optics	1674 reflections with *I* > 2σ(*I*)
graphite	*R*_int_ = 0.023
φ and ω scans	θ_max_ = 30.7°, θ_min_ = 1.6°
Absorption correction: numerical (*SADABS*; Sheldrick, 1996)	*h* = −16→17
*T*_min_ = 0.297, *T*_max_ = 0.675	*k* = −5→5
11800 measured reflections	*l* = −36→37

### Refinement

**Table:**

Refinement on *F*^2^	Primary atom site location: structure-invariant direct methods
Least-squares matrix: full	Secondary atom site location: difference Fourier map
*R*[*F*^2^ > 2σ(*F*^2^)] = 0.019	Hydrogen site location: difference Fourier map
*wR*(*F*^2^) = 0.051	H atoms treated by a mixture of independent and constrained refinement
*S* = 1.03	*w* = 1/[σ^2^(*F*_o_^2^) + (0.0253*P*)^2^ + 1.5219*P*] where *P* = (*F*_o_^2^ + 2*F*_c_^2^)/3
1903 reflections	(Δ/σ)_max_ = 0.002
98 parameters	Δρ_max_ = 0.70 e Å^−^^3^
1 restraint	Δρ_min_ = −0.47 e Å^−^^3^

### Special details

**Table:**

Geometry. All e.s.d.'s (except the e.s.d. in the dihedral angle between two l.s. planes) are estimated using the full covariance matrix. The cell e.s.d.'s are taken into account individually in the estimation of e.s.d.'s in distances, angles and torsion angles; correlations between e.s.d.'s in cell parameters are only used when they are defined by crystal symmetry. An approximate (isotropic) treatment of cell e.s.d.'s is used for estimating e.s.d.'s involving l.s. planes.
Refinement. Refinement of *F*^2^ against ALL reflections. The weighted *R*-factor *wR* and goodness of fit *S* are based on *F*^2^, conventional *R*-factors *R* are based on *F*, with *F* set to zero for negative *F*^2^. The threshold expression of *F*^2^ > σ(*F*^2^) is used only for calculating *R*-factors(gt) *etc*. and is not relevant to the choice of reflections for refinement. *R*-factors based on *F*^2^ are statistically about twice as large as those based on *F*, and *R*- factors based on ALL data will be even larger.

### Fractional atomic coordinates and isotropic or equivalent isotropic displacement parameters (Å^2^)

**Table:**

	*x*	*y*	*z*	*U*_iso_*/*U*_eq_	
Br	0.596053 (15)	1.07921 (4)	0.214841 (6)	0.02877 (6)	
N1	0.54707 (10)	0.7010 (3)	0.06616 (5)	0.0220 (2)	
N2	0.65505 (10)	0.4435 (3)	0.01189 (5)	0.0213 (2)	
H2N	0.5900 (11)	0.412 (5)	−0.0074 (7)	0.026 (5)*	
C1	0.64953 (12)	0.5832 (4)	0.05935 (6)	0.0189 (3)	
C2	0.74416 (12)	0.6124 (4)	0.09880 (6)	0.0205 (3)	
H2	0.8137 (17)	0.536 (5)	0.0930 (8)	0.025 (5)*	
C3	0.73085 (13)	0.7578 (4)	0.14580 (6)	0.0224 (3)	
H3	0.7917 (16)	0.776 (5)	0.1733 (8)	0.026 (5)*	
C4	0.62425 (13)	0.8776 (4)	0.15222 (6)	0.0218 (3)	
C5	0.53646 (13)	0.8461 (4)	0.11167 (6)	0.0231 (3)	
H5	0.4625 (18)	0.923 (5)	0.1152 (9)	0.030 (6)*	
C6	0.7500	0.2511 (5)	0.0000	0.0207 (4)	
H6	0.7804 (16)	0.108 (5)	0.0309 (8)	0.024 (5)*	

### Atomic displacement parameters (Å^2^)

**Table:**

	*U*^11^	*U*^22^	*U*^33^	*U*^12^	*U*^13^	*U*^23^
Br	0.04319 (11)	0.02711 (9)	0.01784 (8)	−0.00538 (7)	0.01056 (6)	−0.00376 (6)
N1	0.0194 (5)	0.0262 (6)	0.0208 (6)	−0.0010 (5)	0.0045 (4)	−0.0038 (5)
N2	0.0166 (5)	0.0286 (6)	0.0188 (6)	−0.0013 (5)	0.0033 (4)	−0.0050 (5)
C1	0.0201 (6)	0.0198 (6)	0.0175 (6)	−0.0032 (5)	0.0052 (5)	0.0006 (5)
C2	0.0192 (6)	0.0231 (7)	0.0192 (6)	−0.0013 (5)	0.0030 (5)	0.0023 (5)
C3	0.0258 (7)	0.0232 (7)	0.0176 (6)	−0.0029 (6)	0.0010 (5)	0.0026 (6)
C4	0.0294 (7)	0.0214 (6)	0.0159 (6)	−0.0049 (5)	0.0074 (5)	−0.0013 (5)
C5	0.0224 (7)	0.0260 (7)	0.0219 (7)	−0.0020 (6)	0.0071 (5)	−0.0032 (6)
C6	0.0227 (9)	0.0204 (9)	0.0199 (9)	0.000	0.0067 (7)	0.000

### Geometric parameters (Å, °)

**Table:**

Br—C4	1.8838 (15)	C2—C3	1.378 (2)
N1—C1	1.3452 (19)	C3—H3	0.94 (2)
N1—C5	1.3356 (19)	C3—C4	1.391 (2)
N2—H2N	0.868 (9)	C4—C5	1.374 (2)
N2—C1	1.3596 (18)	C5—H5	0.95 (2)
N2—C6	1.4425 (17)	C6—N2^i^	1.4425 (17)
C1—C2	1.410 (2)	C6—H6	1.01 (2)
C2—H2	0.92 (2)		
			
C1—N1—C5	118.31 (13)	C2—C3—C4	118.51 (14)
H2N—N2—C1	115.0 (14)	H3—C3—C4	119.9 (13)
H2N—N2—C6	117.5 (14)	Br—C4—C3	122.18 (11)
C1—N2—C6	123.94 (11)	Br—C4—C5	118.90 (12)
N1—C1—N2	115.37 (13)	C3—C4—C5	118.92 (14)
N1—C1—C2	121.41 (13)	N1—C5—C4	123.52 (14)
N2—C1—C2	123.20 (13)	N1—C5—H5	115.8 (13)
C1—C2—H2	120.1 (13)	C4—C5—H5	120.6 (13)
C1—C2—C3	119.31 (14)	N2—C6—N2^i^	114.56 (18)
H2—C2—C3	120.6 (13)	N2—C6—H6	110.2 (11)
C2—C3—H3	121.6 (13)	N2^i^—C6—H6	106.1 (11)
			
C5—N1—C1—N2	−178.95 (14)	C2—C3—C4—Br	179.74 (11)
C5—N1—C1—C2	−0.4 (2)	C2—C3—C4—C5	0.1 (2)
C6—N2—C1—N1	−168.30 (15)	C1—N1—C5—C4	−0.9 (2)
C6—N2—C1—C2	13.2 (2)	Br—C4—C5—N1	−178.55 (12)
N1—C1—C2—C3	1.6 (2)	C3—C4—C5—N1	1.1 (2)
N2—C1—C2—C3	179.98 (14)	C1—N2—C6—N2^i^	−80.54 (14)
C1—C2—C3—C4	−1.4 (2)		

Symmetry codes: (i) −*x*+3/2, *y*, −*z*.

### Hydrogen-bond geometry (Å, °)

**Table:**

*D*—H···*A*	*D*—H	H···*A*	*D*···*A*	*D*—H···*A*
N2—H2N···N1^ii^	0.87 (1)	2.11 (1)	2.9645 (18)	168 (2)

Symmetry codes: (ii) −*x*+1, −*y*+1, −*z*.
